# Similarity in Chronotype and Preferred Time for Sex and Its Role in Relationship Quality and Sexual Satisfaction

**DOI:** 10.3389/fpsyg.2018.00443

**Published:** 2018-04-04

**Authors:** Paulina Jocz, Maciej Stolarski, Konrad S. Jankowski

**Affiliations:** Faculty of Psychology, University of Warsaw, Warsaw, Poland

**Keywords:** chronotype, morningness-eveningness, time for sex, relationship satisfaction, sexual satisfaction, dyadic design

## Abstract

Recently, the issue of diurnal preferences has been increasingly studied within the context of romantic relationships and sexual functioning. In the present paper we apply a dyadic design to investigate the role of romantic partners' diurnal preferences in determining a variety of relationship outcomes. A sample of 91 heterosexual couples completed a set of questionnaires measuring relationship satisfaction, sexual satisfaction, and morningness-eveningness, and answered questions regarding their actual and preferred time for sexual activity. Conducted analyses revealed that similarity in chronotype between partners and female morningness fostered relationship satisfaction in females, but not in males. Furthermore, morningness-eveningness was associated with preferred time for sex in males, but not in females, who in principle preferred evening hours. Although actual time for sex was up to the female preference, sexual satisfaction in both genders was associated with lower discrepancy in their preferred time for sex and greater frequency of intercourse. In sum, these results indicate that chronotype and time for sex are important factors affecting sexual and relationships satisfaction in heterosexual couples.

## Introduction

In the present study we analyze chronotype in context of the satisfaction with functioning in romantic relationships. First we overview the research areas relevant to the study topics; then we specify study aims.

### Chronotype

Morningness-eveningness, also termed chronotype, remains a central dimension of individual differences in the area of biological and psychological circadian rhythms. It is usually treated as a continuous dimension reflecting interindividual variation in diurnal preferences (Natale and Cicogna, 2002). Individuals located on the extremes of this continuum are labeled Morning-types and Evening-types (M-types and E-types), or, more colloquially, larks and owls. However, most people remain in-between; such individuals are labeled Neither-types (N-types; see Adan et al., 2012). Among biological factors, age and gender are related to chronotype. Morningness-eveningness can change over the lifespan. In adolescence a marked shift toward eveningness may often be observed (Díaz-Morales and Randler, 2008; Jankowski, 2015b), whereas adults display a steady shift back to morningness throughout their adult years (Roenneberg et al., 2004; Jankowski, 2015b). Even though gender differences are quite subtle, females usually display higher levels of morningness than males (Randler, 2007). These gender differences are more pronounced for behavioral markers (e.g., sleep timing) as compared to preferences (Jankowski, 2015b), and appear between puberty and the age of 50 years (coinciding with the age of menopause; Roenneberg et al., 2004). The above observations along with gender dimorphism of the circadian system (Bailey and Silver, 2014) suggest a biological basis for such differences.

Individuals with different diurnal preferences vary in several characteristics. Evening chronotypes typically prefer later times of day for their activity, both cognitive and physical; their bed and rise times are naturally later than average and these preferences are associated with a time shift in the course of physiological and psychological rhythms (Goldstein et al., 2007; Adan et al., 2012). Furthermore, E-types compared to M-types displayed a 68-min later peak in the circadian rhythm of body temperature (Bailey and Heitkemper, 2001), lower day-long levels of cortisol (Oginska et al., 2010), higher morning testosterone levels (Randler et al., 2012a), increased vulnerability to a range of health issues (Partonen, 2015), and more symptoms of depression (Jankowski, 2016). In contrast, morningness and earlier sleep timing were related to higher life satisfaction and mood (Jankowski, 2015a).

### Assortative mating

Despite the fact that numerous popular lay theories do not find support in scientific research (Molden and Dweck, 2006), the old adage “birds of a feather flock together” has gained powerful empirical support, at least within the scope of psychological and biological science. Numerous studies have shown that people remaining in long-term relationships tend to display similar levels of various individual differences, and that such congruence results in elevated relationship satisfaction (Gonzaga et al., 2010)—and, consequently, greater relationship longevity (Rammstedt et al., 2013). The magnitude of this within-couple resemblance is often so pronounced that it practically excludes the possibility of random mating, and suggests a methodical, likeness-based process of partner selection. The effect, referred to as assortative mating (Vandenberg, 1972; Buss, 1984, 2003), has gained considerable attention of researchers representing both biological (e.g., Dieckmann and Doebeli, 1999) and psychological science (e.g., Luo and Klohnen, 2005).

As Smieja and Stolarski (2018) summarize, “positive assortative mating (similarity; also labeled as “homogamic mating”) is indicated by a positive correlation between male and female partners' scores on the same characteristic, while negative assortative mating (complementarity; heterogamic mating) is indicated by scores being correlated negatively” (1). Systematic partner selection, also labeled initial assortment, seems to be the most important process underpinning assortative mating effect (Keller et al., 1996); however, a convergence phenomenon, manifested in increasing between-partner similarity over time, is also possible (albeit less common; see Gonzaga et al., 2010). Assortative mating remains a universal phenomenon, occurring “across different species, and with respect to various characteristics” (Smieja and Stolarski, 2018, (2). As Keller et al. (1996) note, certain individual differences regularly yield greater similarity effects than others (see Watson et al., 2004 for an overview). The highest positive assortment effects, often oscillating around the level of 0.70 or even 0.80, are found for sociodemographic variables such as age, social class, educational level, and attitudes (Feng and Baker, 1994; Domanski and Przybysz, 2007; Escorial and Martín-Buro, 2012). In the case of cognitive abilities and IQ, assortative mating effects are not so pronounced; however, they still remain meaningful, with correlations ranging between 0.30 and 0.60 (Colom et al., 2002; van Leeuwen et al., 2008). With respect to personality traits, the magnitude of assortative mating effect is strongly differentiated; estimates vary across particular studies and between various traits (see Luo and Klohnen, 2005; Gonzaga et al., 2010). For instance, Watson et al. (2004) reported rather low levels of within-couple similarity for personality dimensions. Escorial and Martín-Buro (2012) suggested that assortment for personality is of modest magnitude, whereas other researchers obtained associations exceeding the level of 0.40 (e.g., McCrae et al., 2008).

### Chronotype and mating

Recently, the issue of assortative mating for diurnal preferences has been studied within the context of romantic relationships and sexual functioning. Randler and Kretz (2011) were first to demonstrate assortative mating effect for morningness-eveningness. In their study the convergence between partners' chronotype amounted to 0.55 (0.40 after controlling for age). Following this line of research, in two studies with females reporting the timing of their own and their partners activities, there was a modest similarity between partners in sleep times, e.g., mid-sleep on free days (Leonhard and Randler, 2009; Randler et al., 2014). Thus, the magnitude of the abovementioned assortative mating effects for chronotype resembles the effects obtained for personality traits.

Furthermore, in long-term relationships, women would prefer their partners to be more synchronized with their own diurnal preferences, i.e., shifted toward morningness (Randler et al., 2014). As for short-term mating, eveningness in males has been related to a higher number of sexual partners (Piffer, 2010; Gunawardane et al., 2011; Randler et al., 2012b)—what could be treated as an indicator of greater mating success. These effects could be partially explained by higher levels of testosterone in evening males than in morning males (Randler et al., 2012a). Further studies, however, showed that evening preference is associated with a tendency to engage in uncommitted sexual relations also in females. This effect can be observed not only on the behavioral level, but also in desire and positive attitude toward uncommitted sex (Jankowski et al., 2014b; Randler et al., 2016).

Circadian variation can also be found in sexual activity. Two major peaks of sexual encounters have been revealed in a study on fifteen university students (Refinetti, 2005). A dominant peak occurred between 11:00 p.m. and 1:00 a.m., while the second, minor peak took place between 6:00 a.m. and 8:00 a.m. Importantly, participants explained that the main reason for their choice was partner availability (around bedtime). Similar conclusions were drawn from a study of 78 young married couples: the researchers reported a major peak in sexual activity in the evening, and another minor peak in the morning (Palmer et al., 1982). In a study of 135 female university students (Barak et al., 1997), the evening intensification of sexual activity proved even more pronounced: 85% of participants declared that they lost virginity in the evening/night hours. Morningness-eveningness, however, appears to influence rather the timing of desire for sex than the actual undertaking of sexual activity: the latter typically occurs between 9:00 p.m. and 12:00 a.m., regardless of chronotype (Jankowski et al., 2014a). Consequently, the timing of desire is positively, but only modestly, associated with the actual timing of sexual activity (Jankowski et al., 2014a).

### Relationship quality

Hitherto studies indicated various personality aspects potentially influencing relationship quality. The meta-analysis by Malouff et al. (2010) revealed significant effects of Neuroticism (−0.22), Extraversion (0.06), Agreeableness (0.15), and Conscientiousness (0.12) on relationship satisfaction. The latter personality dimension was one of the most marked personality correlates of morningness (Hogben et al., 2007). Factors other than personality can also influence both relationship quality and sexual satisfaction. One of the major determinants of the former is constructiveness of communication between partners (Litzinger and Gordon, 2005). Further, according to the Interpersonal Exchange Model of Sexual Satisfaction (Lawrance and Byers, 1995), sexual satisfaction in long-term relationships is determined by: (1) one's level of rewards gained and costs incurred in the relationship; (2) one's own comparison standards for rewards and costs; as well as (3) an individual's perception of within-dyad equality of those rewards and costs.

### Study aims

The present study had three aims:

1. The first aim was to test the importance of similarity in daily functioning between partners in heterosexual romantic relationships. Specifically, we were interested in the role of similarity in preferred time for sleep and activity (i.e., chronotype) and time of day for undertaking sexual activity in sexual and relationship satisfaction. We hypothesized that within-couple convergence in these variables leads to greater satisfaction (both in terms of relationship and sexual life). The reasoning behind this hypothesis is as follows: (1) individual differences in preferred time for sex do exist and are at least partly associated with individual differences in chronotype (Jankowski et al., 2014a); (2) some couples are mismatched in terms of chronotypes and people, in general, would prefer their partners to be to more similar to themselves in terms of diurnal preferences (Randler et al., 2014); and (3) activities undertaken during optimal times of day are performed more efficiently. This observation, known as synchrony effect and first shown for cognition (May, 1999), we assume to exist in other activities relevant for relationship quality. Specifically, we claim that similarity in chronotype between partners creates “common temporal space” which facilitates various activities important for relationships, such as communication (Litzinger and Gordon, 2005) and sexual activity. Similarity in chronotype and consequent “common temporal space” may result in higher frequency and quality of joint activities (including sex-related ones), as the phases of internal motivation for these actions would appear simultaneously in both partners. Consequently, the relationship satisfaction should be higher, as the activity (e.g., sexual intercourse) would result from internal motives of both partners, not only from the willingness to satisfy their partner's needs. What is more, one can imagine how being mismatched in chronotypes or preferred time for sex may influence the balance of profits and losses and, indirectly, partners' sexual satisfaction. As an example, adjusting time for undertaking sexual activity to the partner's preferences may result in lower satisfaction (e.g., “we have sex only when my partner wants; my preferences do not count”). On the other hand, having sex with a partner who forces him/herself to make love solely to respond to the other's need can be also uncomfortable and result in a sense of guilt. Therefore, it seems justified to expect that diurnal preference and its within-couple composition may influence a variety of relationship outcomes at many different levels.
  In summary, based on the above arguments, it seems reasonable to expect that a mismatch in chronotype may lead to a mismatch in preferred time for sex. Such a temporal discrepancy in preferred time for sex may lead to an impression of discrepancy in sexual desire, which is known to affect both sexual satisfaction and, in consequence, relationship satisfaction (Davies et al., 1999).
2. The second aim of this study was to test whether assortative effects found previously for chronotype (Randler and Kretz, 2011) can also be observed for preferred time for sex. This issue has not been studied to date and, given the importance of sexual activity for relationship satisfaction, one may argue that people do not pair based on chronotype itself, but rather on preferred time for sex (associated with it).
3. The third aim was to test the expectation that there is a positive effect of morning preference on relationship satisfaction. It is possible that M-types more positively judge various aspects of their life, including relationship satisfaction. This effect has been observed in previous studies for life satisfaction (Jankowski, 2015a) and depressive symptoms (Jankowski, 2016). E-types, on the other hand, are characterized by cognitive negative biases in emotional processing (Berdynaj et al., 2016).

### Novelty of the study

In summary, the present study attempts to answer three novel questions: (1) whether a non-random assortment with respect to preferred time for sexual activity exists; (2) whether the degree of between-partners similarity in morningness-eveningness and preferred time for sex influences perceived satisfaction with the relationship; and (3) whether morningness predicts satisfaction with relationship. Combining the issue of similarity with satisfaction is crucial here: building upon the results of Luo and Klohnen (2005) showing that similarity in personality fosters relationship quality, we add to the field by testing the effect for diurnal preferences. Thus, the present study advances the field of chronopsychology, focusing on the consequences of diurnal rhythms for the quality of relationship/sexual functioning. It also extends knowledge of the underpinnings of relationship satisfaction and sexual satisfaction.

## Materials and methods

### Participants

Participants were 91 heterosexual Polish couples (91 females and 91 males) aged between 18 and 38 (*M* = 25.96, SD = 3.66) and being a couple for at least 6 months. Relationship length ranged between 6 and 192 months (16 years), with a mean of 53.4 months (i.e., around 4.5 years), and SD of 39.3 months (3.3 years). Most couples were either married and living together (34.1%), non-married and living together (58.3%), or non-married and sleeping together (17.6%). Most of the couples had no children (81.1%). Participants were mostly residents of cities of 400,000 and more inhabitants (62.8%) with higher education (60.22%). Among the participants 13.7% were E-types, 72.5% were N-types, and 13.7% were M-types.

### Procedure

The study took place in Warsaw, Poland. The sample was recruited by a specially trained pollster who invited participants using social media and personal connections. To take part in the study, both partners had to declare that they have had sex with each other for at least 6 months. These inclusion criteria were emphasized by our pollster during the recruitment procedure. To ensure the criteria were met, we asked about: (1) relationship length, and (2) time since the first sexual intercourse with the present partner on the first page of our questionnaire booklet. Each couple was tested individually in a separate room selected for the purposes of the present study. Participants completed the set of measures in the presence of a pollster, who took care to prevent any communication between partners. Partners were also not allowed to compare results with each other. Participants were not rewarded. Written informed consent was obtained from all participants of the present study.

**Morningness-eveningness preference** was measured with the Composite Scale of Morningness (CSM; Smith et al., 1989; Jankowski, 2015b). CSM is a 13-item self-report questionnaire applying a Likert-type response format with either four or five response options. Greater morningness is indicated by higher scores, whereas greater eveningness is indicated by lower scores. The bottom 10% of distribution in a large sample obtained 23 points or less (E-types), while the top 10% obtained 43 points or more (M-types) (Jankowski, 2015b). In the present study internal consistency of the scale, measured with Cronbach α, was 0.91, both in females and males.

**Sexual satisfaction** was assessed with the Index of Sexual Satisfaction (ISS; Hudson et al., 1981). This self-report one-scale inventory contains 25 items (e.g., “Our sex life is very exciting,” or “My partner seems to avoid sexual contact with me”), which are rated on a 5- or 7-point Likert-type scale, depending on the version of the questionnaire. In this study, we used a 7-point scale, ranging from 1 (*none of the time*) to 7 (*all of the time*). Originally, ISS was developed for clinical use, to measure the level of sexual discord or dissatisfaction in one's relationship with their partner, so higher scores indicated greater degree of sexual discord. In the present study, we used an inverse answer coding, so that higher scores indicated greater sexual satisfaction. This alteration was made to simplify the interpretation results. Psychometric properties of the Polish version of the questionnaire (see Stolarski et al., 2016) are high (i.e., Cronbach's alpha = 0.94; correlations with intercourse frequency Spearman's rho = 0.57 for both men and women in validation studies in Poland; in the present sample, Cronbach's alpha amounted to 0.92 for men and 0.93 for women).

General **relationship satisfaction** was measured with the Relationship Assessment Scale (RAS; Hendrick, 1988). This 7-item self-report scale is one of the most commonly used measures in the research of relationship quality. Degree of one's (dis)agreement with each of the items is rated using a 5-point Likert-type scale ranging from 1 (*low satisfaction*) to 5 (*high satisfaction*). Psychometric properties of the Polish version of the questionnaire are good, with Cronbach's alpha of 0.88 and substantial correlations with other relationship quality indicators (see Stolarski et al., 2016). In the present study, the high internal consistency of the measure was confirmed (Cronbach's alpha amounted to 0.85 for males and 0.87 for females), as was the scale's convergent validity (intercorrelations with ISS amounted to 0.62 in males and 0.66 in females).

The optimal and actual **time of day for sex** was assessed as in a previous study (Jankowski et al., 2014a). Specifically, two questions (“What time of day do you usually want to have sex most” and “What time of day do you usually undertake sexual activity”) with a single choice response format were used (time intervals: between 12:00 to 3:00 a.m.; 3:00 to 6:00 a.m., 6:00 to 9:00 a.m., 9:00 a.m. to 12:00 p.m., 12:00 to 3:00 p.m., 3:00 to 6:00 p.m., 6:00 to 9:00 p.m., and 9:00 p.m. to 12:00 a.m.). None of the participants selected the 3:00–6:00 a.m. period in any of the two questions. Therefore, we treated this interval as a boundary between extreme morning and extreme evening preferences/behaviors. Both these variables were then analyzed as continuous variables with the 6:00 to 9:00 a.m. time interval indicating extreme morning and 12:00 to3:00 a.m. indicating extreme evening. To enable correlational analyses and mean comparisons, each of the time intervals was ranked from 1 (6:00 to 9:00 a.m.) to 7 (12:00 to 3:00 a.m.), and the novel variable was treated as a continuous indicator of time for sex. For the presentation of means and SDs, the numeric variable was again transformed into hh:mm coding to make the statistics easier to read. The declarations of typical time for sexual intercourse revealed only a moderately high consistency between partners (*r* = 0.58, *p* < 0.01).

**Demographic variables** included gender, age, education (incomplete primary, primary, incomplete secondary, secondary, student, higher), place of residence (village, city of under 100,000 inhabitants, city of more than 100,000 inhabitants but under 400,000 inhabitants, city of 400,000 or more inhabitants), relationship status (non-formal relationship and living apart, non-formal relationship and living apart but often having sleepovers, non-formal relationship and living together, married); relationship duration (length of relationship in years and months; the answers were later transformed into months); frequency of intercourse (“typical” month frequency, without specific situations like longer absence of one of the partners); and having children. Although the frequency of intercourse is an objective value, slight differences between partners in exact average numbers of intercourse per month may appear. In our sample partners were highly consistent in their declarations (*r* = 0.86, *p* < 0.001); we decided to take an average value of both partners' declarations for further analyses.

## Results

**Figure 1:**
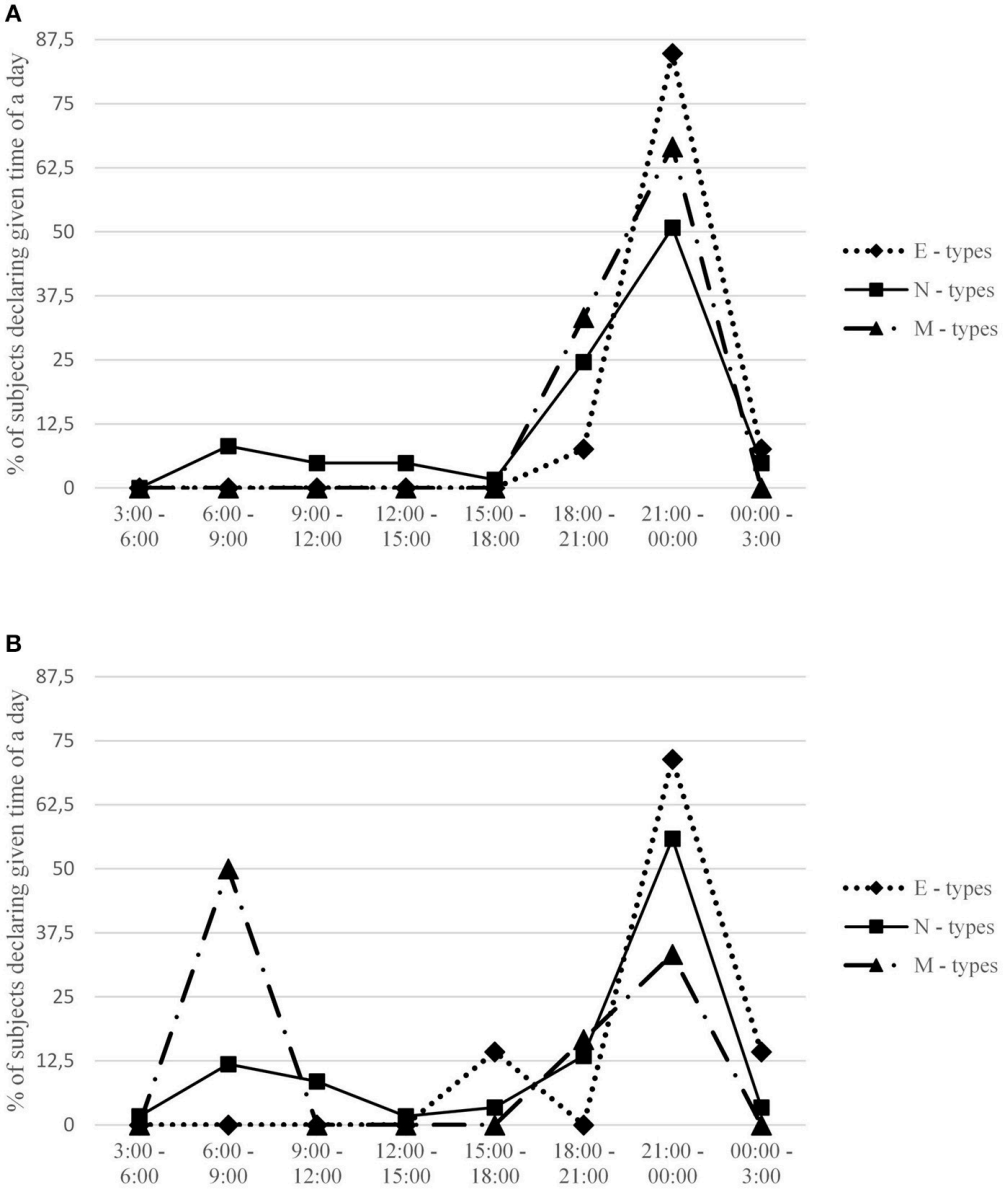
Percentage of subjects in each chronotype group declaring a given time interval when they desired sex the most. Females **(A)** and males **(B)** are presented separately. Expected percentage assuming no circadian variation is 12.5 for each time interval.

**Figure 2:**
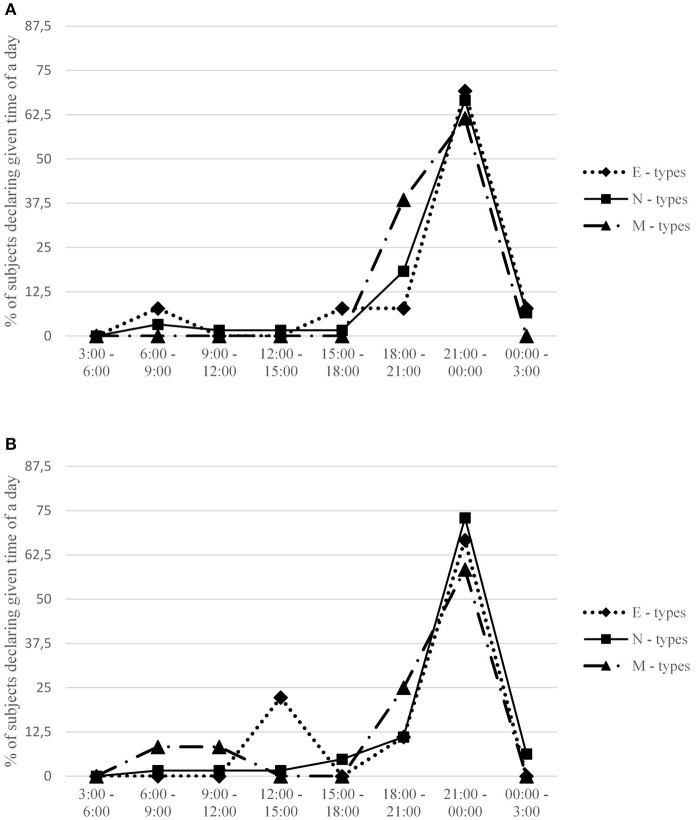
Percentage of subjects in each chronotype group declaring a given time interval when they usually undertook sexual activity. Females **(A)** and males **(B)** are presented separately. Expected percentage assuming no circadian variation is 12.5 for each time interval.

**Figure 3:**
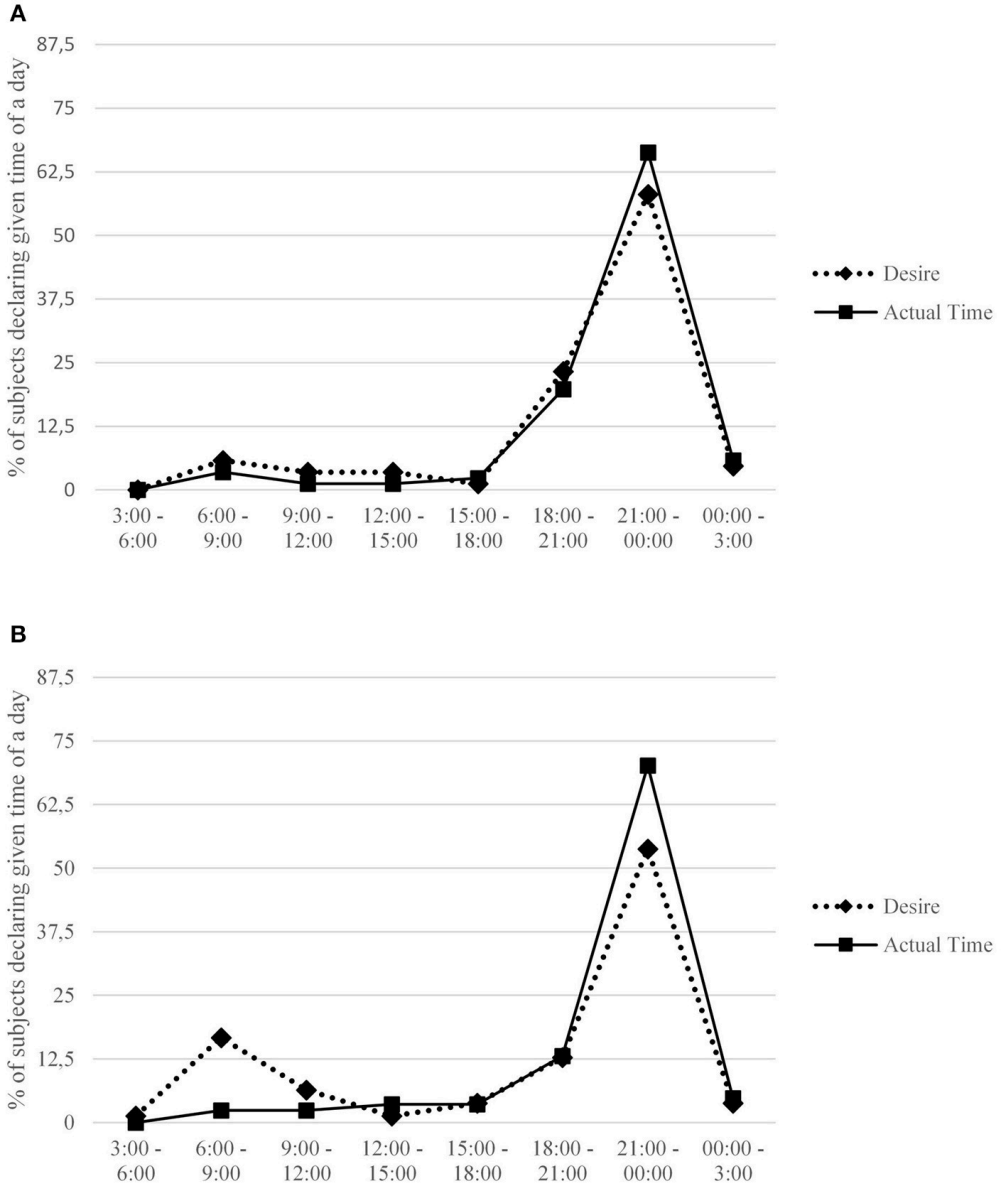
Percentage of subjects in total declaring a given time interval when they desired sex the most and usually undertook sexual activity. Females **(A)** and males **(B)** are presented separately.

Descriptive statistics and intercorrelations between measured variables are provided in Table 1. Detailed information regarding preferred and actual time for undertaking sexual activities for each gender and across particular chronotypes is presented in Figures 1, 2. A comparison between preferred and actual time for sex is presented for each gender in Figure 3.

**Table 1:** Descriptive statistics and intercorrelations between measured variables.

	**M**	**SD**	**α**	**1**	**2**	**3**	**4**	**5**	**6**	**7**	**8**	**9**	**10**	**11**	**12**	**13**	**14**
**MALE**																	
1. Morningness-eveningness	34.98	7.99	0.91	–													
2. Sexual Satisfaction	5.99	0.69	0.92	−0.04	–												
3. Relationship Satisfaction	4.33	0.57	0.85	−0.06	0.62^**^	–											
4. Preferred time for sex	18:23	6:13	–	−0.36^**^	0.10	0.13	–										
5. Actual time for sex	21:04	3:34	–	−0.14	0.45^**^	0.32^**^	0.14	–									
6. Preferred vs. actual (abs. diff.)	4:12	5:53	–	0.18	−0.07	−0.30^**^	−0.73^**^	−0.20	–								
**FEMALE**																	
7. Morningness-eveningness	33.88	7.97	0.91	0.09	0.17	0.19	0.04	−0.01	−0.11	–							
8. Sexual Satisfaction	6.10	0.72	0.93	−0.14	0.66^**^	0.46^**^	0.21	0.44^**^	−0.12	0.18	–						
9. Relationship Satisfaction	4.29	0.59	0.87	−0.17	0.42^**^	0.51^**^	0.17	0.31^**^	−0.21	0.28^**^	0.66^**^	–					
10. Preferred time for sex	20:17	4:23	–	−0.06	−0.04	0.10	0.05	0.27^*^	−0.29^*^	−0.12	0.13	0.07	–				
11. Actual time for sex	21:11	3:27	–	−0.05	0.08	0.16	−0.04	0.59^**^	−0.24^*^	0.01	0.13	0.17	0.40^**^	–			
12. Preferred vs. actual (abs. diff.)	1:59	3:52	–	0.04	0.15	0.07	−0.20	−0.06	0.18	−0.06	0.01	−0.06	−0.55^**^	−0.32^**^	–		
**COUPLE**																	
13. Preferred (M) vs. preferred (F) (abs. diff.)	4:39	6:04	–	0.24^*^	−40^**^	−36^**^	−64^**^	−39^**^	0.71^**^	−0.13	−0.32^**^	−0.2^*^	−0.23^*^	−0.24^*^	0.03	–	
14. Morningness-eveningness (abs. diff.)	8.33	6.85	–	0.14	−0.19	−0.15	−0.17	−0.04	0.17	−0.18	−0.04	−0.29^**^	0.19	0.12	−0.02	0.19	–
15. Frequency of intercourse	10.29	6.46	–	−0.08	0.45^**^	0.28^**^	0.05	0.24^*^	−0.04	0.04	0.41^**^	0.24^*^	0.10	0.10	0.04	−0.18	−0.04

* *p < 0.05*,

** *p < 0.01. Preferred, preferred time for sex; actual, actual time for sex; abs. diff., absolute value of the difference*.

First, it should be noted that relationship satisfaction and sexual satisfaction were robustly intercorrelated both in males (*r* = 0.62, *p* < 0.01) and in females (*r* = 0.66, *p* < 0.01). Partners were also quite consistent in their assessments of satisfaction: the between-partner correlations amounted to *r* = 0.66, *p* < 0.01, for sexual satisfaction, and *r* = 0.51, *p* < 0.01, for general relationship satisfaction. Finally, frequency of intercourse was associated both with males' (*r* = 0.45, *p* < 0.01) and females' (*r* = 0.41, *p* < 0.01) sexual satisfaction, and somewhat less with relationship satisfaction (*r* = 0.28, *p* < 0.01 and *r* = 0.24, *p* < 0.01, respectively).

Contrary to our hypotheses, the correlations indicating assortative mating for chronotype (see also Table 2) and preferred time for sex were not apparent (*r* = 0.09, ns., and *r* = 0.05, ns., respectively) and also when controlling for relationship length (*r* = 0.03, ns., and *r* = 0.05, ns.). Moreover, the difference between partners' chronotypes and preferred time for sex did not prove to change with relationship length (*r* = 0.04, ns., and *r* = 0.06, ns., respectively), showing that no convergence effects could be observed for these variables.

**Table 2:** Distribution of men and women within the couples according to their chronotypes.

	**Males**		
**Females**	**E-types (*N* = 11)**	**N-types (*N* = 68)**	**M-types (*N* = 12)**
E-types (*N* = 14)	3	9	2
N-types (*N* = 64)	8	48	8
M-types (*N* = 13)	0	11	2

Males scoring high on morningness declared that they preferred to have sex in earlier hours than evening males (*r* = −0.36, *p* < 0.01), providing some evidence for consistency between chronotype and sexual drive in this group. This effect is visible also in Figure 1: 50% of male M-types declared that they have the highest desire for sex between 6:00 and 9:00 in the morning, whereas over 70% of evening types would prefer to have sex late in the evening, between 9:00 p.m. and midnight.

An analogical effect was not observed in females, whose most desired time for sex was unrelated to their morningness-eveningness. Again, the effect is also visible in Figure 1: both M-type and E-type females prefer to have sex in the evening (although the former prefer to have it before 9:00 p.m., whereas the latter prefer after 9:00 p.m.). The gender difference is also clearly visible in the marked difference between males and females in mean preferred time for sex (see Table 3).

**Table 3:** Between-gender mean comparisons.

	**Females**		**Males**		**t**	**g^†^**
	**M**	**SD**	**M**	**SD**		
Morningness-eveningness	33.88	7.97	34.98	7.99	0.97	−0.14
Sexual satisfaction	6.09	0.72	5.99	0.69	−1.58	0.14
Relationship satisfaction	4.28	0.59	4.33	0.57	0.81	−0.09
Preferred time for sex	20:20	4:17	18:16	6:14	−2.42^*^	0.38
Actual time for sex	21:07	3:34	21:05	3:36	−0.10	0.01
Preferred vs. actual (difference)	1:59	3:50	4:21	5:56	3.14^**^	−0.47

* *p < 0.05*,

** *p < 0.01*.

† *Hedges' effect size measure. Some of the means presented in the present table may differ from those reported in Table 1 due to some missing data points in one or other gender. Mean values for preferred and actual time for sex were calculated based on the assumption that each time period (e.g., 21:00–24:00) is represented by its center (i.e., 22:30)*.

Interestingly, actual time for sex was related only to females' preferred time for sexual activity, and not to the time indicated by males (see Table 1). Furthermore, the between-partner difference in preferred time for sex was greater in couples with a more morning-oriented male (*r* = 0.24, *p* < 0.05). Given that the difference in preferred time for sex was a negative predictor of satisfaction in both genders (in males: *r* = −0.40, *p* < 0.01, for sexual satisfaction, and *r* = −0.36, *p* < 0.01 for general satisfaction; in females, respectively, *r* = −0.32, *p* < 0.01 and *r* = −0.24, *p* < 0.05), this effect may indicate that morning chronotype in males has an indirect negative effect on satisfaction, via generating difference in preferred time for sex (females generally prefer sex in the evening, see Table 1; in males it depends on diurnal preference; see Table 3). Interestingly, although the discrepancy in preferred time for sex was clearly associated with relationship quality (see Table 1), analogical correlation for the frequency of intercourse did not reach statistical significance.

Although inconsistencies in preferred time for sex were robustly associated with relationship satisfaction, analogical effects were not observed for inconsistencies in morningess-eveningness preference, with an exception for females' relationship satisfaction (which proved negatively associated with this discrepancy).

Finally, we observed that morning-oriented females are generally more satisfied with their relationship than their evening-oriented ones, whereas in males no association between chronotype and satisfaction was observed.

## Discussion

In the present study we attempted to investigate the role of romantic partners' diurnal preferences in determining a variety of relationship outcomes using dyadic design, where a couple remains a unit of analyses. The main findings are that: (1) similarity in chronotype between partners and female morningness fostered relationship satisfaction in females, but not in males; (2) morningness-eveningness was associated with preferred time for sex in males, but not in females, who in principle preferred evening hours; (3) actual time for sex was up to the female preference; and (4) sexual satisfaction in both genders was associated with lower discrepancy in their preferred time for sex and greater frequency of intercourse. The main study outcomes are discussed below in more detail.

First, it should be noted that, unlike in previous studies (Randler and Kretz, 2011; Randler et al., 2014), we have not obtained any evidence for assortative mating for chronotype or preferred time of day for sexual activity. This result could seem surprising, given that previous studies provided evidence for marked assortment effects for chronotype and indicators of sleep timing (Randler and Kretz, 2011; Randler et al., 2014). The inconsistency between studies could result, however, from differences in relationship duration—a relatively short length in our sample (4.5 years compared to 15.6 years in Randler and Kretz, 2011 sample) and age of participants—relatively young in our sample (26 years compared to 39 years in Randler and Kretz, 2011 sample). Even though Randler and Kretz (2011) found a correlation between partners' chronotype also when controlling for relationship duration, the mentioned discrepancies between the studies trigger a hypothesis that assortative mating for chronotype occur in older, more committed relationships, but not for the younger ones. This hypothesis could be tested by comparing participants with presumably different levels of commitment, e.g., people in marriages vs. people in informal relationships. The hypothesized effect of commitment on assortative mating has been previously observed for some variables other than chronotype (Blackwell and Lichter, 2000).

The mismatch in chronotype was related to relationship satisfaction, but only in females. This is in line with previous observations that, for committed relationships, females prefer partners who would be more in sync with their chronotype (Randler et al., 2014). Given that males have later sleep times compared to females (Jankowski, 2015b), it means that females may not prefer evening-oriented partners for long term relationships. In other words, it appears that females have different preferences for males' chronotype depending on their actual focus on long- vs. short-term relationships. Females who seek for a partner for a short-term relationship, seem to prefer evening males—such a conclusion could be derived from the results showing that E-type men report greater mating success understood as higher number of female sexual partners, also those being in a relationship with another man (Randler et al., 2012b).

Morningness was associated with preferred time for sex in males, but not with actual time of sex. In females, no such effect was observed, probably due to a much lesser difference between preferred and actual time for sex in females who, regardless of their chronotype, prefer to have sex in the evening. The latter effect may be interpreted in light of gender differences in erotic plasticity (Baumeister, 2000). According to Baumeister (2004), “female sexuality is inherently more amenable than male sexuality to influence by cultural events, historical circumstances, socialization, peer influence, and other social variables” (133); as a result, it remains much less biological. The effect should also be observable in preferences for sexual activity, for which evening seems to be a “culturally approved” time of day. Consequently, females follow cultural norms regarding the optimal time of day for sexual behaviors, whereas males remain more biologically-determined in their sexuality, including preferences driven by diurnal biological rhythms. Unlike in previous research (e.g., Larson et al., 1991), we have not observed the association between chronotype mismatch and intercourse frequency. This may be also caused by relatively young age and short relationship length in the present sample.

These gender differences are even more interesting if we look at the associations of both chronotype and preferred time for sex with actual time of undertaking sexual activity, which proved unrelated to both these variables in males. On the other hand, the actual time for sex proved associated with females' preferred time for sex. It is worth noting that the main gender difference between patterns of desire for sex pertains to the morning: hardly any females identified morning hours as the most desired time for sex, while in males this choice was much more popular. Thus, although male M-types would eagerly have sex in the morning, the actual time of their sexual activity has little to do with their preferences; it depends mainly on preferences of their female partners. According to Lykins et al. (2006), females generally report decreased sexual interest when they are in a bad mood whereas in males the desire for undertaking sexual practices is more independent of their actual affective states. Moreover, males' sexual desire is considered more frequent and stronger than females' (see Leiblum, 2002; Baumeister, 2004). As a result, males may be more willing to make concessions regarding actual time for sex than females, agreeing to have sex in the time indicated by their female partners. Consequently, females' preferences become a decisive factor underpinning actual time for intercourse, whereas males' preferences have little to do with it.

Finally, both analyzed types of satisfaction proved markedly associated with the discrepancy between partners in preferred time for sex. This result provides a clear evidence that synchrony in diurnal preferences may robustly influence the quality of functioning in romantic relationships. Interestingly, the discrepancy was not significantly associated with frequency of intercourse. Thus, it seems that a lack of consistency in preferred time for sex can influence the quality, as opposed to the quantity, of sexual interactions. Further, factors other than low frequency of sexual activity mediate these associations. Thus, sexual motivation seems sufficiently powerful to foster sexual behaviors, regardless of whether both partners or only one of them preferred having sex in the particular moment. However, coupling in times not considered optimal by both partners results in lower level of sexual satisfaction and, probably as a result, in decreased overall relationship satisfaction.

Although morningness-eveningness proved to be associated with a variety of relationship outcomes, the frequency of sexual intercourse proved generally unrelated to partners' diurnal preference and its composition within couples. This suggests that some other factors play a dominant role in determining this aspect of functioning in romantic couples. Major determinants of intercourse frequency include: biological aging, health quality, habituation to sex, relationship satisfaction, pregnancy, and presence of small children (see Call et al., 1995 for a review). Morningness-eveningess is associated with many of these variables, and their often powerful effects on intercourse frequency may actually suppress the effects of diurnal preference. For instance, morningness is positively associated with both age and health (see Adan et al., 2012), whereas these two outcomes have opposite effects on intercourse frequency (Call et al., 1995). In-depth analyses conducted on greater and more differentiated samples could allow for: (1) controlling for such effects; and (2) uncovering eventual effects of diurnal preference and partner similarity in chronotype on the quantitative aspect of sexual functioning.

## Limitations and future directions

The main limitation of this study concerns the small number of studied couples. This results in some statistically non-significant results (these eventually could become significant if the sample were larger, provided the effect sizes were not lowered). This applies, for instance, to the correlation between male morningness-eveningness and actual time for sex: the coefficient would be statistically significant at *p* < 0.05 for 200 couples resulting in 400 participants. One hundred eighty-two participants comprising 91 couples in our sample did not allow proving statistically significant correlations weaker than 0.2. It must be noticed though that, due to methodological factors related to studying romantic couples, such a sample size is not substantially smaller compared to other dyadic studies (e.g., Zeidner and Kaluda, 2008; Stolarski et al., 2011; Lamkin et al., 2015).

Moreover, our sample comprised mainly young couples, both in terms of age of partners and relationship length, and only heterosexual couples were taken into account. Most were inhabitants of big agglomerations (poorer couples, as well as those with lower levels of education, members of the working-class, or military couples were strongly underrepresented). These facts limit generalizability of the present results. Future studies, therefore, should seek to analyze not only larger, but also more differentiated, samples.

In particular, studies focusing separately on comparisons between couples with and without children could provide some interesting insights into the nature of associations reported in our research. Although in the present sample there were no significant differences in relationship and sexual satisfaction, frequency of sexual intercourse between married couples and non-married couples living together, or between couples with vs. without children, it would certainly be valuable to establish whether the present results are replicated in each of these samples. Taking into account the potential moderating role of social status of the participating couples (e.g., replicating the results in a sample derived solely from working-class or military couples), could be also informative in terms of determining generality vs. specificity of the present results. Finally, as sexual preference was shown to have a powerful impact on many relationship outcomes and could act as a moderator of associations well-established in the research on romantic dyads (see Mark et al., 2015), replications of the present analyses on samples differing in sexual orientation could also bring some valuable insights into the nature of the obtained associations.

Furthermore, the present study has all limitations characteristic of cross-sectional research. Longitudinal analyses would enable conclusions about actual causality of the demonstrated associations. Such studies would be particularly interesting, as they would additionally allow identification of previously unexplored predictors of relationship dissolution (see Le et al., 2010).

Finally, it would also be interesting to seek out potential mediators of the obtained associations. Chronotype is associated with a broad spectrum of dimensions that may influence relationship quality, such as emotional intelligence (Stolarski and Jankowski, 2015) and sociosexuality (Jankowski et al., 2014b). Taking them into account would provide some insight into the nature of effects identified in the present study.

Although while developing the present study we had no ambitions to provide any practical solutions guide, some interesting conclusions for psychological and sexological practice can be derived based on the outcomes of our research. The impact of the composition of diurnal preferences regarding time for sexual intercourse is not an obvious factor to be considered by psychotherapists and sexologists. It therefore may easily be neglected. The present study provides some evidence that discrepancies in preferred time for sex may result in some undesirable consequences for the quality of sexual activity in couples. Thus—at least in couples divergent in terms of chronotypes and preferred time for sex—considering these discrepancies may prove important for proper understanding of the causes of eventual dissatisfaction. Taking them into account may turn out to be an important step in developing effective interventions or suggesting changes in sexual behaviors that could improve the quality of sexual functioning in such dyads.

## Ethics statement

This study, including the consent process, was approved by the ethics committee of Faculty of Psychology at University of Warsaw. Informed written consent was obtained from all participants. Participation was voluntary and participants were allowed to reject or withdraw at any point with no disadvantage to their treatments.

## Author contributions

PJ and MS: designed the study; PJ: conducted the study; PJ and MS: conducted the analyses; PJ: described the analysis; PJ, MS, and KJ: wrote the manuscript.

### Conflict of interest statement

The authors declare that the research was conducted in the absence of any commercial or financial relationships that could be construed as a potential conflict of interest. The reviewer, MŻ, and handling Editor declared their shared affiliation.
